# Management of serious complications in intra‐abdominal desmoid‐type fibromatosis

**DOI:** 10.1002/cnr2.1411

**Published:** 2021-06-24

**Authors:** Federica Bini, Marco Fiore, Salvatore Provenzano, Rossella Bertulli, Arianna Ottini, Chiara Colombo, Marco Vitellaro, Gabriella Greco, Carlo Morosi, Alessandro Gronchi, Paolo Giovanni Casali, Elena Palassini

**Affiliations:** ^1^ Postgraduation School in Medical Oncology Università Politecnica delle Marche Ancona Italy; ^2^ Oncological Surgery Unit 4, Department of Surgery Fondazione IRCCS Istituto Nazionale dei Tumori Milan Italy; ^3^ Medical Oncology Unit 2, Medical Oncology Department Fondazione IRCCS Istituto Nazionale dei Tumori Milan Italy; ^4^ Postgraduation School in Medical Oncology Università degli Studi di Milano Milan Italy; ^5^ Hereditary Digestive Tract Tumors Unit, Department of Surgery Fondazione IRCCS Istituto Nazionale dei Tumori Milan Italy; ^6^ Diagnostic and Interventional Radiology Fondazione IRCCS Istituto Nazionale dei Tumori Milan Italy; ^7^ Oncology and Haemato‐Oncology Department University of Milan Milan Italy

**Keywords:** cancer management, chemotherapy, clinical outcome, medical oncology, rare cancer, sarcoma

## Abstract

**Background:**

Desmoid fibromatosis (DF) is a rare and locally infiltrative monoclonal fibroblastic proliferation arising from connective tissues, with lack of metastatic potential. About 10% of all DF cases are intra‐abdominally sited. Complications in this site, due to the locally infiltrative nature of the disease, may be severe and potentially life threatening. However, data on incidence, management, and outcome of these complications are limited.

**Aim:**

Data of patients with sporadic or FAP‐related intra‐abdominal DF treated at Istituto Nazionale dei Tumori (INT) in Milano from 2005 to 2020 who developed a serious complication during the course of their disease were retrospectively collected and analyzed with a descriptive statistics.

**Methods and Results:**

Out of 72 intra‐abdominal DF, 8 cases were identified (M/F: 5/3, median age: 35 years, FAP‐related/sporadic: 2/6): 3 with bowel obstruction, 5 with bowel perforation. In 4 cases the serious complication was the first evidence of disease; in the other 4 cases it occurred at a time interval from diagnosis in the range of 4–44 months (during an active surveillance program in one case and during chemotherapy in the other 3 cases). A surgical treatment was feasible and successful in 5 cases. In 3 surgically unmanageable patients, all progressing and experiencing acute complications while on chemotherapy, a non‐surgical approach with intensive supportive treatment and with a prompt change of chemotherapy regimen was implemented, being successful in two, the other patient dying due to a concomitant progressive lymphoma thereafter.

**Conclusion:**

In this series of intra‐abdominal DF, the incidence of serious complications was 11%. Most patients were successfully treated with surgery. When surgery was deemed to be unfeasible, a conservative management with intensive supportive care and a careful choice of chemotherapy was adopted, ensuring a favorable outcome in most.

## 1 INTRODUCTION

Desmoid‐type fibromatosis (DF), also known as desmoid tumor or aggressive fibromatosis, is a monoclonal fibroblastic proliferation arising from connective tissues, which lacks any metastatic potential but has a tendency to be locally infiltrative.^1^, ^2^ With an incidence of 2–4 cases per million per year, DF is a rare disease, accounting for about 3% of all soft tissue tumors,^3^ with a peak incidence between 25 and 35 years of age and a higher prevalence in females.^4^ In 85%–90% of cases, the disease is sporadic and it is characterized by the presence of a somatic inactivating mutation in CTNNB1 gene.^5^ A germline APC gene mutation is harbored in the remaining 10%–15% of DF cases, resulting in the Gardner's syndrome phenotype, a subtype of the familial adenomatous polyposis (FAP).^5^ Patients with FAP have a life‐long risk of about 10%–15% to develop DF, resulting in a risk 850 times greater than in the general population.^3^

DF can occur in any anatomical location. In the sporadic setting, the disease is most commonly located to girdles, extremities and abdominal wall. In these sites, the locally infiltrative nature of the disease may result in functional impairment and pain, with a significant impact on quality of life, but low risk of death. Intra‐abdominal‐site is rare in sporadic setting, accounting for less than 5%–10% of cases, but is very common in FAP‐related cases, accounting for 80% of cases.^6^ Overall, intra‐abdominal site accounts for about 10% of all DF. In this site, given the locally aggressiveness of the disease, several severe complications with a potential risk of death may occur, including bowel obstructions, perforations, and ischaemia.^7^, ^8^ In FAP patients treated with a prophylactic colectomy, DF is the leading cause of death, with a mortality rate of 10%.^9^, ^10^ Nevertheless, since DF is rare and uncommonly intra‐abdominally located, data on incidence, management, and outcome of these complications in patients with intra‐abdominal DF are limited.

On this basis, we retrospectively selected patients with sporadic or FAP‐related intra‐abdominal DF treated at the Fondazione IRCCS Istituto Nazionale dei Tumori (INT), Milan, Italy, over 15 years, and reviewed healthcare records of patients who experienced a serious complication.

## 2 PATIENTS AND METHODS

The medical records of all consecutive patients with sporadic or FAP‐related intra‐abdominal DF treated from 2005 to 2020 at INT who experienced a serious complication during the course of their disease were retrospectively reviewed. The following data were collected: time of occurrence of the serious complication from diagnosis of DF; treatment on‐going at the onset of the serious complication; type of serious complication, management, and outcome. Details on DF treatment strategy adopted before and after the serious complication were also recorded. Data collected included also gender, age, DF size, and multifocality. Follow‐up was updated as of July 2020. Descriptive statistics were used. This study was approved by the institutional Ethics Committee.

## 3 RESULTS

Seventy‐two patients with intra‐abdominal DF were treated at INT over the study period: 51 with mesenteric DF and 21 with retroperitoneal or pelvic DF. In 61 cases the disease was sporadic and in 11 cases it arose in the context of FAP. Out of these 72 patients, 8 patients (11%), who experienced a serious complication, were identified for this study. All 8 cases had mesenteric DF, so that the incidence of serious complication was 16% when only mesenteric DF was considered. Two cases had FAP‐related DF (18% of all FAP cases) and 6 had sporadic disease (10% of all sporadic cases). Three patients experienced a bowel obstruction and 5 patients had a bowel perforation. Three patients were females and 5 were males. The median age at the onset of the event was 35 years (range 20–56 years). The median maximum diameter of the lesion at the time of the event was 18 cm (range 6–35 cm). Three patients had multifocal intra‐abdominal disease.

In 4 patients, the diagnosis of DF followed the serious complication and the radiological evidence of an intra‐abdominal lesion. In the other 4 cases, the serious complication occurred after 4, 5, 6, and 44 months from diagnosis, respectively. In particular, in one case the serious complication occurred during an active surveillance program and in the other 3 cases it occurred during chemotherapy (at 1 week, 1 and 4 months from the start of chemotherapy, respectively).

In 5 cases, the management of the serious complication was based on a surgical treatment (palliative surgery, palliative surgery followed by complete surgery, macroscopic intralesional surgery, and complete surgery respectively in 2, 1, 1, and 1 cases). Two FAP patients were treated with a palliative surgery after the onset of a small‐bowel obstruction and the CT evidence of an intra‐abdominal lesion. After the histological confirmation of DF diagnosis, both patients received a low‐dose chemotherapy with methotrexate and vinorelbine (MTX‐VA), for 40 and 43 cycles, respectively, achieving a dimensional response. Ileostomy was removed after 1 year from the withdrawal of chemotherapy in both cases. Another patient was initially treated with a palliative surgery after the CT evidence of an intra‐abdominal lesion causing a small‐bowel perforation. A complete surgery with ileostomy removal was performed 6 months later. A further patient, initially managed elsewhere with active surveillance because asymptomatic, was treated with a macroscopic intralesional surgery after 5 months from the diagnosis, following the evidence of a progression of the disease, causing a small‐bowel perforation. Subsequently, because of the presence of a macroscopic residual disease, the patient received a low‐dose chemotherapy, achieving a dimensional shrinkage. One additional patient was treated with a complete surgery after the onset of fever, abdominal pain and the radiological evidence of an intra‐abdominal mass causing large‐bowel perforation. After 30 months from the event, in this last case, a CT scan showed a local relapse and a treatment with pazopanib was started. Treatment was on‐going at the moment we were writing this report. After a median follow‐up of 41 months (range 25–59 months) all patients are alive, 4 of them free from progression, one with an event of local relapse.

**FIGURE 1:**
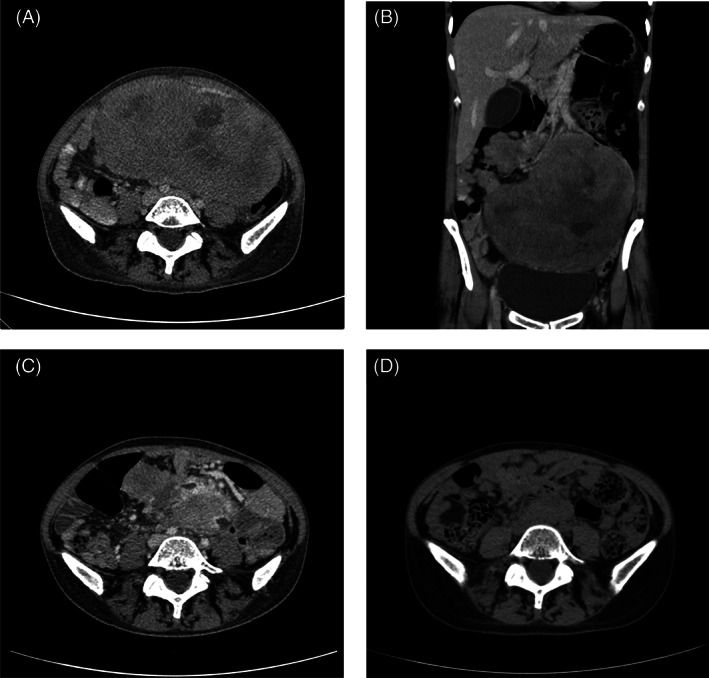
Case 1. (A,B) Contrast‐enhanced CT scan, at the onset of severe complication. (C) After 5 weekly cycles with MTX‐VA. (D) After a total of 42 cycles with MTX‐VA [Correction added on 12 July 2021, after first online publication: The size of Figures 1 and 2 has been updated in this version].

**FIGURE 2:**
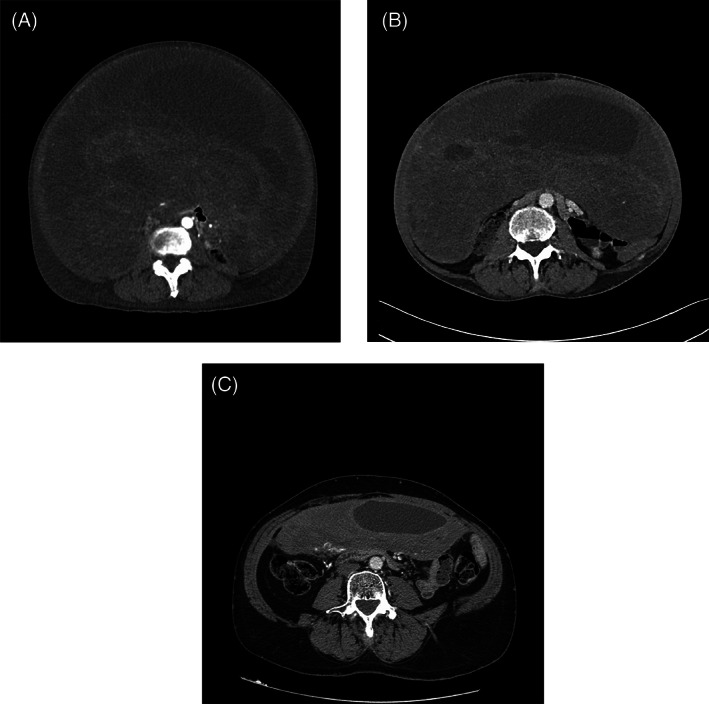
Case 3. (A) Contrast‐enhanced CT scan, at the onset of severe complication (35 × 22 cm). (B) DF shrinkage (30 × 12 cm) after 14 weekly cycles of doxorubicin at the discharge. (C) The last CT scan showing further reduction of the DF (23.5 × 6.5 cm), 6 months after the discontinuation of the chemotherapy [Correction added on 12 July 2021, after first online publication: In the original published version, Figures 1 and 2 have been corrected the size in this version].

A conservative management of the serious complication was adopted in 3 cases, because a surgical treatment was deemed to be unfeasible. These 3 cases are detailed hereafter. Details about chemotherapy doses administered are reported in Table 1. CT images related to cases 1 and 3 are shown in Figures 1 and 2, respectively.

**TABLE 1:** Chemotherapy regimens

Case	Chemotherapy		
1	Liposomal doxorubicin (50 mg/sqm)	Doxorubicin + Dacarbazine (75 mg/sqm + 800 mg/sqm)	Methotrexate + Vinorelbine (50 mg + 20 mg/sqm)
2	RCHOP Protocol (Rituximab 375 mg/sqm IV + Vincristine 1.4 mg/sqm IV + Doxorubicin 50 mg/sqm IV + Cyclophosphamide 750 mg/sqm IV + Prednisolone 100 mg)	Doxorubicin (20 mg/sqm)	R‐DHAOx Protocol (Dexamethasone 40 mg IV/PO + Rituximab 375 mg/sqm IV + Oxaliplatin 65 mg/sqm IV + Cytarabine 1000 mg/sqm IV)
3	Methotrexate+Vinorelbine (50 mg + 20 mg/sqm)	Doxorubicin (25 mg/sqm)	

4

### 4.1 Case 1

After a diagnosis of mesenteric DF (10 cm), this patient (49 years, female), who was asymptomatic, was seen at INT in April 2012. An active surveillance program was started and a progressive dimensional shrinkage (up to 7 cm) was observed until August 2015, when, following a dimensional increase of the lesion (up to 11 cm) with the appearance of a new satellite lesion (4.5 cm), a liposomal doxorubicin‐based chemotherapy was started. After rapid progression of disease (with an increase of lesions up to 19 and 6 cm, respectively), conditioning bowel sub‐obstruction with fever, the patient was admitted to INT on September 18, 2015. Antibiotic and analgesic therapy were implemented and one cycle of doxorubicin plus dacarbazine was administered after 2 weeks. A few weeks later, following an episode of severe acute anemia and abdominal pain, radiological signs of duodenal and small bowel perforation were detected (Figure 1A,B). A surgical approach was deemed not to be feasible and the patient was treated with a conservative approach. After recurrent intestinal bleeding episodes, low‐dose chemotherapy with MTX‐VA was started, 47 days after the hospitalization. Symptomatic improvement was obtained in the following weeks and an initial reduction in size of the lesions (13 and 5 cm) was observed after 5 weekly cycles (Figure 1C). The patient was discharged after almost 3 months of hospitalization. Low‐dose chemotherapy with MTX‐VA was continued on an outpatient basis, for a total of 42 cycles, until December 2016, obtaining a progressive reduction in size of the lesions (6 and 3.5 cm) (Figure 1D). In January 2020, after 51 months from the event of bowel perforation, the patient was free from progression and asymptomatic.

### 4.2 Case 2

This patient (40 years, female), who was asymptomatic, came to our attention in October 2019, after the diagnosis of mesenteric DF (9.5 cm). Low‐dose chemotherapy with MTX‐VA was recommended. Because of a subsequent diagnosis of follicular lymphoma, 3 months later a treatment with R‐CHOP (rituximab, cyclophosphamide, doxorubicin, vincristine, and prednisone) was started. After rapid worsening of clinical condition, with acute abdominal pain, on January 30, 2020 the patient was admitted to INT. CT scan showed a tumor response as far as lymphoma was concerned, but a dimensional increase (19 cm) of the DF lesion was observed, with signs of bowel perforation. A surgical treatment was excluded, and a conservative management was undertaken. A doxorubicin‐based treatment was continued during hospitalization, with weekly administration of doxorubicin for three cycles. Symptomatic improvement was obtained and the patient was discharged after 2 months of hospitalization. Since the following CT showed a shrinkage of the DF lesion (16 cm) but an increase of mediastinal and abdominal lymph nodes, R‐CHOP was resumed, with evidence of further progression after two cycles. A rescue chemotherapy with rituximab, oxaliplatin, and cytarabine (R‐DHAOx) was started, but after one cycle further lymphoma progression and dimensional increase of DF lesion (18 cm) were observed. Best supportive care was selected and 8 months after the onset of the severe complication this patient died.

### 4.3 Case 3

In October 2018, this patient (male, 57 years), who was asymptomatic, came for an outpatient visit at INT, after being diagnosed with intra‐abdominal DF (20 cm) a few weeks earlier. An active treatment with low‐dose MTX‐VA was proposed. On March 29, 2019, the patient was again evaluated at INT, after 12 weekly cycles of chemotherapy. General condition appeared worsened, with symptoms of bowel sub‐obstruction and malnutrition, and the patient was admitted. A CT scan showed a dimensional increase of the lesion (up to 35 × 22 cm) (Figure 2A) with bilateral pleural effusion. A surgical treatment was excluded, and a conservative management was implemented together with the prompt administration of weekly doxorubicin, starting 4 days after the hospitalization. Symptomatic improvement was observed during the following weeks. One month after the hospitalization, pulmonary thromboembolism (PTE) was diagnosed and therapy with anticoagulants was started, with resolution of the event. After 4 months of hospitalization, having received 14 weekly cycles of doxorubicin with symptomatic and radiological response (30 × 12 cm), the patient was discharged. Administration of weekly doxorubicin was continued on an outpatient basis, up to October 2019, for a total of 24 cycles, obtaining further lesion shrinkage (27 × 9.5 cm) (Figure 2B). Few weeks after the withdrawal of the chemotherapy the patient was diagnosed with a congestive heart failure and a medical therapy was started with rapid improving of myocardial function. On April 2020, the patient was in good general condition, with a CT scan showing further reduction of the abdominal mass (23.5 × 6.5 cm), 6 months after the discontinuation of the chemotherapy and 13 months after the onset of this serious complication (Figure 2C).

## 5 DISCUSSION

Out of 72 patients with intra‐abdominal DF treated at INT over the last 15 years, the incidence of a serious complication was 11%, in terms of bowel obstructions or perforations, which in 4 cases were the first evidence of disease. A surgical treatment was feasible and successful in 5 cases, while a non‐surgical approach was used in 3, being successful in two, the other patient dying due to a concomitant progressive lymphoma thereafter.

In this retrospective case series analysis of exclusively intra‐abdominal DF patients treated at a reference Center over 15 years, the number of serious complications was low, but not negligible, especially in comparison to extra‐abdominal DF and abdominal wall DF. Clearly, intra‐abdominal DF is a rare condition, so that one can speculate even on such a limited number of cases. Indeed, the incidence was 11% of all patients, but 16% when only mesenteric DF is considered. One should be aware that DF is a non‐metastasizing disease, with an overall very good prognosis in terms of life expectancy. Even in this series, none of our patients died to the complication (one of them died early after, but the cause was a concomitant progressive non‐Hodgkin's lymphoma).

The importance of complications in intra‐abdominal DF is due to the increasingly widespread conservative approach to DF in general. Up to some years ago, surgery was viewed as standard treatment in DF. Today, front‐line surgery is generally not of choice, given the high risk of local recurrences even after adequate resections and the high rate of functional sequelae at several sites.^1^, ^11^, ^12^ Specifically, in intra‐abdominal DF, the reported recurrence rate after surgery is in the 20%–65% range in available retrospective series.^13^, ^14^, ^15^, ^16^, ^17^ Moreover, depending on its peculiar mesenteric location, intra‐abdominal DF is poorly manageable with reasonably conservative surgery in a substantial proportion of patients. Besides, serious peri‐operative complications may be in the 15% range.^18^ Thus, also in intra‐abdominal DF a front‐line non‐surgical approach is recommended, in terms of active surveillance and medical therapy as needed, and the role of surgery may then be largely confined to patients undergoing local‐regional complications.^11^ This was the case with 5 of our complicated patients, who were managed surgically. In 3 of them, complete surgery was not feasible, and the intervention was palliative or intralesional, a medical treatment being subsequently offered.

Our approach was non‐surgical in three patients, because any surgery was deemed to be unfeasible. All three patients experienced acute complications during chemotherapy, as a consequence of progressive disease. Intensive supportive treatment was administered, along with antibiotic therapy, and a careful change of chemotherapy regimen was established soon after the stabilization of the acute condition. The outcome was favorable in two patients, the other dying of a concomitant non‐Hodgkin's lymphoma after 8 months. A good tumor response was achieved with an anthracyline‐based chemotherapy in one of them (after progression to low‐dose chemotherapy) and vice versa with low‐dose chemotherapy in the other (after progression to anthracyclines). Both regimens are known to be active in DF.^5^, ^19^, ^20^, ^21^, ^22^ Since a tumor response may be achieved earlier with anthracyclines, an anthracycline‐based regimen could be preferred in a setting where prompt tumor shrinkage is important. However, the better hematological toxicity profile of low‐dose chemotherapy may be useful in a critical patient. In theory, anti‐tyrosine kinase inhibitors (e.g., sorafenib or pazopanib) could be preferred in terms of response rate, but clearly antiangiogenics are generally problematic in acute abdominal presentations, due to risks of bleeding and suboptimal tissue repair.

Non‐surgical policies in DF basically mean active surveillance or medical therapy. Active surveillance is based on the reported number of long‐term disease stabilizations, and even spontaneous regressions, without any treatment.^21^ Regressions in the absence of any treatment were observed also in 20% of intra‐abdominal DF.^14^ Over‐treatment is thus avoided for indolent tumors. Medical therapy is generally offered to progressive and/or symptomatic patients.^1^, ^23^, ^24^, ^25^, ^26^ In this sense, intra‐abdominal DF should be approached with more caution, due to the need to balance the risks of over‐treatment with the risks of serious complications in case of rapid progression. Thus, there is consensus about possibly earlier decisions of active treatment in intra‐abdominal DF.^1^ An effort to define criteria thereof would be worthwhile.^27^ In any case, careful monitoring of patients allocated to active surveillance is required. One should notice that, even if very rare, progression may follow a spontaneous regression, as occurred in one of our patients. Moreover, patient referral to a sarcoma Centre of expertise is always recommended from the time of diagnosis.

In conclusion, a policy of active surveillance is tenable also in abdominal DF, as in DF in general, but the clinician needs to be aware that serious complications are relatively more common. When surgery is not resorted to even at the time of any such complications, intensive supportive care and an accurate choice of chemotherapy may well be resorted to.

## CONFLICT OF INTEREST

The following represents disclosure information provided by authors of this manuscript. All relationships are considered compensated unless otherwise noted. Relationships are self‐held unless noted.

F.B., M.F., A.O., C.C., M.V., G.C., C.M.: No competing interests to declare.

S.P., R.B., E.P.: Institutional research funding Amgen Dompé, Bayer, GlaxoSmithKline, Novartis, Pfizer, PharmaMar, Eisai, Eli Lilly, Advenchen Lab, AROG Pharmac, Blueprint Medicines, Deciphera, Epizyme, Karyopharm Pharmac, Daiichi Sankyo.

A.G.: Honoraria from Novartis, Pfizer, Eli Lilly, PharmaMar; consulting or advisory role for Novartis, Bayer, Eli Lilly, Pfizer, Nanobiotix, PharmaMar, SpringWorks; research funding from PharmaMar (Inst), PharmaMar, Nanobiotix.

P.G.C.: Consulting or advisory Role for Bayer, Deciphera; speakers' bureau for Pfizer; institutional research funding from Amgen Dompé, Bayer, GlaxoSmithKline, Novartis, Pfizer, PharmaMar, Eisai, Eli Lilly, Advenchen Lab, AROG Pharmac, Blueprint Medicines, Deciphera, Epizyme, Karyopharm Pharmac, Daiichi Sankyo.

## AUTHOR CONTRIBUTIONS

All authors had full access to the data in the study and take responsibility for the integrity of the data and the accuracy of the data analysis. *Conceptualization*, E.P. and F.B.; *Methodology*, E.P.; *Investigation*, E.P., A.O., and F.B.; *Formal Analysis*, E.P. and F.B.; *Resources*, M.F., G.G., and C.M.; *Writing ‐ Original Draft*, E.P., F. B.; *Writing ‐ Review & Editing*, F.B., E.P., S.P., R.B., C.C., M.V., A.G., and P.G.B.; *Supervision*, P.G.C. The corresponding author attests that all listed authors meet authorship criteria and that no others meeting criteria have been omitted.

## ETHICS STATEMENT

This study was approved by the Ethical Committee of IRCCS Istituto Nazionale dei Tumori (reference number INT 191/20). All participants provided written informed consent. This study was performed in accordance with the Declaration of Helsinki.

## Data Availability

The data that support the findings of this study are available from the corresponding author upon reasonable request.
